# Parents’ knowledge, attitude and practice towards children’s vaccination in Lebanon: role of the parent-physician communication

**DOI:** 10.1186/s12889-020-09526-3

**Published:** 2020-09-22

**Authors:** Perla Matta, Rayane El Mouallem, Marwan Akel, Souheil Hallit, Marie-Claude Fadous Khalife

**Affiliations:** 1 Faculty of Medicine and Medical Sciences, Holy Spirit University of Kaslik (USEK), Jounieh, Lebanon; 2 Pediatrics and Neonatology Department, Notre Dame Des Secours University Hospital, Byblos, Lebanon; 3 INSPECT-LB: Institut National de Santé Publique, Epidémiologie Clinique et Toxicologie-Liban, Beirut, Lebanon; 4 School of Pharmacy, Lebanese International University, Beirut, Lebanon

**Keywords:** Immunization coverage, Knowledge, Attitude, Physician-patient communication, Lebanon

## Abstract

**Background:**

One of the most effective public health interventions in the world is immunization. However, some parents doubt its usefulness and safety. Many factors influence their decision to vaccinate, including their sociodemographic characteristics, their trust in the public health system, the parent-physician relationship, their level of knowledge and their attitudes towards vaccination. Our objective was to determine the factors, especially the parent-physician communication, associated with parental knowledge, attitudes and practices of their children’s vaccination.

**Methods:**

Three thousand five hundred parents (father, mother or both) of children aged between 1 month and 15 years were approached by a trained personnel who performed the data collection through personal interviews (February–April 2019).

**Results:**

The response rate was 79.5%. The results of the multivariable analysis showed that a better patient-physician communication was significantly associated with higher knowledge, better attitude and practice. Better knowledge was significantly associated with better attitude, whereas better knowledge and attitude were significantly associated with better practice.

**Conclusion:**

Our study shows the importance of good physician-patient communication in improving knowledge, attitude and practice of parents towards their children’s vaccination.

## Background

One of the most important discoveries in medicine is vaccination. It is one aspect of public health that is considered the most cost-effective in reducing the prevalence of life-threatening and contagious diseases [1]. Immunization is believed to save between 2 and 3 million lives each year [2]. The concept of immunization is not limited to one person but concerns the community as a whole: a vaccinated child is not only protecting himself but also others by preventing the transmission of vaccine preventable diseases (VPD). This is known as herd immunity [3].

A decrease in multiple VPD has been noticed for multiple years [4], with an increase in the number of unvaccinated children reported recently. One study done in the US mentioned that the number of parents who refused to vaccinate their children doubled between the years 2006 and 2013 [5]. Furthermore, the refusal of vaccination has become very common among parents globally, which has led to the increase in the rates of certain diseases, like measles. In 2018, according to the World Health Organization (WHO), almost 20 million children were not vaccinated against “measles, diphtheria and tetanus” [6]. In 2017, 1.5 million children died from VPD [4].

In Lebanon, we have witnessed an increased number of VPD, especially measles and mumps, since the arrival of refugees due to the emergence of poor sanitary conditions and the presence of anti-vaxxers among the population [7]. The percentage of children vaccinated against measles (measles containing vaccine MCV) in 2018 was well below the recommended 95% (82% for MCV1 and 63% for MCV2). These factors have led to the outbreak of measles and mumps and have made the elimination goal more difficult to achieve [8]. On another note, some vaccines in Lebanon are considered voluntary like those against rotavirus and hepatitis A and are usually not covered by the ministry of public health. This leads to the high rate of transmission of these infectious diseases [9].

WHO defines vaccine hesitancy as the refusal or delay in vaccination [10]. The major reason for this behavior is the doubts about the safety of the vaccines, fueled by bad experiences or by media [11]. The easy access of the internet nowadays has helped anti-vaccination campaigns reach more people and has facilitated the spread of misinformation [12]. Another reason for hesitancy is the infrequent observance of the negative outcomes of VPD as they have become relatively rare. Because of this, many parents believe that vaccines are unnecessary, and that the harms outweigh the benefits [5, 13].

Regarding vaccination practices, many factors contribute to the decision-making process. First of all, multiple studies have shown that unvaccinated children were mostly white, had older mothers with higher levels of education and were of families of high income [14, 15]. Other studies concluded that mothers who were more educated tended to vaccinate their children more [9, 16]. The cost of the vaccines seems to be one of the determinants of the immunization status [17]. Moreover, it has been proven in many studies that living with people who support immunization and vaccinate their children resulted in positive attitudes towards vaccination [18].

Second, the trust in the health-care system and the relationship with the pediatrician or physician are important determinants of the attitudes towards vaccines. As a matter of fact, the more trust the parents have in the several health institutions, the more knowledge they acquire about the benefits and risks of vaccines [19, 20]. The type of relationship between the parent and the physician has shifted through the years and has become based nowadays on communication and shared decisions [21]. Many parents find themselves lacking knowledge about the concept of vaccination and start looking elsewhere when there is poor communication with the pediatrician, often stumbling upon myths and false information [22].

Furthermore, the level of knowledge of parents is an essential determinant of their practices. Knowledge directly affects attitude, thus, working on educating parents should be a basis for acquiring better attitudes and practices [23]. Many talked about the association between the lack of immunization and the lack of knowledge regarding vaccine necessity [24]. Conversely, others talked about how parents who have less knowledge about immunization were more compliant. This was explained by the fact that parents who acquired knowledge about vaccines also questioned their safety and necessity [25].

In addition, another factor related to immunization practices is the parental attitude towards vaccines. Benin and colleagues split parents into groups regarding their views on vaccination: “rejecters”, “late vaccinators”, “vaccine-hesitant” and “accepters” [26]. Studies have shown that mothers who had negative attitudes towards vaccination didn’t vaccinate their children and didn’t attempt to gain knowledge about immunization [27].

Health authorities in Lebanon have been raising the alarm for the past few years on the importance of vaccination after the outbreaks of infectious diseases that were previously controlled. Despite the efforts put into trying to raise the rate of vaccinated children, Lebanon is still far from reaching the worldwide-recommended rates. Our study objectives were to assess factors associated with parental knowledge, attitudes and practices (KAP) of children vaccination among parents in Lebanon. To our knowledge, few studies [9, 28] conducted in Lebanon tackled some factors but none discussed the association between the patient-physician communication and the KAP triad. In these studies done in 2016 and 2018, some of the factors that were assessed were parental age, level of education, vaccine price and family size [9, 29]. However, since vaccination rate in Lebanon is still below the 95% target set by WHO, our results would help understand the factors influencing the decision to vaccinate and developing actions or measures in order to improve immunization practices and adherence in Lebanon. We will include in our paper the methods used to collect the data, the questionnaires involved, and the statistical analysis applied. Then we will discuss our results and compare them with those found in the literature.

## Methods

### Study design

**Fig. 1:**
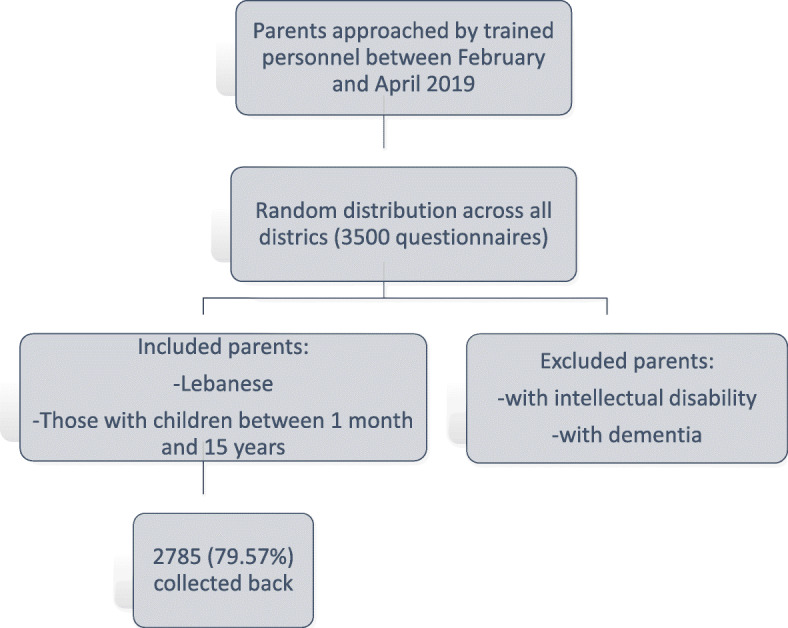
Flow chart summarizing the study design

This is a national cross-sectional study that was conducted between February and April 2019; 3500 printed copies of the questionnaire were distributed randomly to multiple villages proportionately in all Lebanese districts. Simple randomization was used to pick the villages. Participants were enrolled in a proportionate way from each region according to the number of persons living in each district according to the Central Agency of Statistics in Lebanon. Parents of children (fathers, mothers or both) aged between 1 month and 15 years were invited to fill the survey by trained personnel, who performed data collection through personal interviews with the parents. Excluded were those who refused to fill the questionnaire and those with intellectual disability and dementia, as reported by a family member (Fig. 1). Persons involved in the data collection had training before launching data collection to ensure the quality of research and avoid interrater variability as much as possible.

### Minimal sample size calculation

In the absence of similar studies in Lebanon, taking an expected percentage of parental knowledge about children vaccination of 50%, a design effect of 2 and an error of 5%, the minimal sample size calculated was estimated at 768 using the Epi-info software.

### Questionnaire

The questionnaire was in Arabic. The items included were derived from surveys carried out previously to understand the level of parental knowledge regarding vaccination, their attitudes, their practices and their relationship with the physician [30–32].

It was made up of four parts: the first part dealt with the socio-demographic characteristics of the parent: sex, age, marital status, level of education, monthly income, number of rooms in the house, number of persons living in the same house, number of children, and third-party payers. The second part assessed the level of knowledge of parents about vaccination, the way they acquire their information, and the level of trust granted to the: pediatrician, family doctor, pharmacist, friends, social networks and the ministry of public health. This part included items derived from previous surveys [33–35], with response options comprising “yes”, “no” or “I do not know” for some items with a minimum score of 0 and a maximum score of 2 for each question, and a 5-point Likert scale ranging from “agree” to “totally disagree” for the others with a minimum score of 1 and a maximum score of 5. The third part concerned parents’ attitudes towards vaccines, including their confidence in vaccines, the factors associated with the choice of vaccines, their opinion on their usefulness, effectiveness and safety. The items included in this part were also extracted from previous surveys [33, 34, 36]. The patient-physician communication (PPC) scale consisting of 15 items was added to the questionnaire [33]. This tool was used to evaluate the effect of the PPC on the parents’ adherence to the vaccination schedule. A 5-point Likert scale ranging from “agree” to “totally disagree” was used in this part with a minimum score of 1 and a maximum score of 5 for each question. The final part evaluated parents’ vaccination practices: whether they are up to date, if they ever refused or missed a vaccine, and if they ever had side effects. Response options that were used comprised of “yes”, “no” or “I do not know” with a minimum score of 0 and a maximum score of 2 for each question. All other scales used were a 5-point Likert scale ranging from “agree” to “totally disagree” with a minimum score of 1 and a maximum score of 5 for each question. Higher scores indicated better KAP and PPC respectively. Finally, the three scores were divided, using the visual binning option in SPSS, into three categories each to divide the participants into having poor, moderate and good knowledge/attitude/practice. The total score range of knowledge varied between 2 and 32, that of attitude between 10 and 50, and that of practice between 0 and 12.

### Forward and back translation method

One healthcare professional carried out the translation from English to Arabic. This forward translation was then translated by a second healthcare professional back to Arabic. No major differences were found between the two English versions, with discrepancies resolved by consensus. A pilot test of the Arabic version was performed on 20 parents, before launching data collection. The pilot sample’s results were included in the final data sheet.

### Statistical analysis

Data analysis was conducted using SPSS software version 23. Weighting to the general population was performed regarding gender, age, and governorate of dwelling. The Kolmogorov-Smirnov and Shapiro-Wilk tests were used to verify the normal distribution of the sample; therefore, parametric tests were used. The Student t-test and ANOVA test were used to compare two and three or more means respectively, whereas two continuous variables were compared using the Pearson’s correlation test. Since better knowledge implies an enhanced attitude, which leads to an improved practice, three hierarchical stepwise linear regressions were conducted. The first linear regression considered knowledge as the dependent variable (DV) and the sociodemographic variables and PPC as independent variables (IVs). The second linear regression considered attitude as the DV, with the sociodemographic variables, PPC and knowledge as IVs The third regression considered practice as the DV and the sociodemographic variables, PPC, knowledge and attitude as IVs. IVs included in those regressions were those that had a *p* < 0.1 in the bivariate analysis. Scales’ reliability was assessed using Cronbach’s alpha. *P* < 0.05 was considered significant.

## Results

A sensitivity analysis was done to check for interrater variability, no difference was detected between them. Therefore, the whole database was considered as one set. The Cronbach’s alpha values for the scales were as follows: knowledge (α = 0.633), attitude (α = 0.624), practice (α = 0.539) and PPC (α = 0.925).

### Sociodemographic and other characteristics of the participants

Out of 3500 questionnaires, 2785 (79.57%) were collected back. The sociodemographic and other characteristics of the participants are summarized in Table 1 (mean age: 40.14 ± 13.45; 35.2% males). Moreover, the means and SD for the scales scores were as follows: knowledge (18.10 ± 3.83), attitude (41.21 ± 4.91), practice (10.82 ± 2.70) and patient-physician communication (64.44 ± 9.12).

**Table 1:** Description of the sample’s characteristics

Variable	N (%)
**Gender**	
Male	955 (34.3%)
Female	1757 (63.1%)
**District**	
Beirut	839 (30.3%)
Mount Lebanon	565 (20.4%)
North	726 (26.2%)
South	565 (20.4%)
Bekaa	84 (3.0%)
**Education level**	
Illiteracy	41 (1.5%)
Primary	192 (7.1%)
Complementary	437 (16.1%)
Secondary	658 (24.2%)
University	1387 (51.1%)
**Marital status**	
Married	2683 (96.3%)
Single (divorced/widowed)	102 (3.7%)
**Monthly salary**	
< 1000 USD	988 (39.5%)
1000–2000 USD	1051 (42.1%)
> 2000 USD	460 (18.4%)
**Type of insurance**	
None	789 (29.1%)
National Social Security Fund (NSSF)	1115 (41.2%)
Army	352 (13.0%)
Private	326 (12.0%)
Cooperative	127 (4.7%)
	**Mean ± SD**
Age (in years)	40.14 ± 13.45
House crowding index	1.12 ± 0.64
Number of children	2.92 ± 1.50

Moreover, 1154 (41.4%) parents had poor knowledge (scores ≤17), whereas 874 (31.4%) and 757 (27.2%) had moderate (scores between 18 and 20) and good (scores of 21 and above) knowledge respectively. On another hand, 955 (34.5%) parents had poor attitude (scores ≤39), whereas 1070 (38.7%) and 740 (26.8%) had moderate (scores between 40 and 44) and good (scores of 45 and above) attitude respectively. Finally, 989 (35.7%) parents had poor practice (scores ≤10), whereas 875 (31.5%) and 910 (32.8%) had moderate (scores between 10.01 and 12) and good (scores of 12.01 and above) practice respectively.

### Bivariate analysis

Bivariate analyses were performed to determine relationships between knowledge, attitude and practice, and other variables pertaining to the participants. A significantly higher mean knowledge score was found in females compared to males, in those living in South Lebanon compared to all other districts, in those having a university level of education compared to all other categories, in married parents compared to single status people (divorced/widowed), in employees compared to low and intermediate ones and in those having a cooperative insurance compared to all other categories.

A significantly higher mean attitude score was found in females compared to males, and in those having a university level of education compared to all other categories. Furthermore, married parents and those having a cooperative insurance had a higher mean attitude score.

A significantly higher mean practice score was found in females compared to males, in those having a university level of education compared to all other categories, in married parents compared to those with a single status (divorced/widowed) and in those having a cooperative insurance compared to all other categories (Table 2).

**Table 2:** Bivariate analysis of sociodemographic and other factors associated with the knowledge, attitude and practice scores

Variable	Knowledge	Attitude	Practice
**Gender**			
Male	17.65 ± 3.95	40.70 ± 5.17	10.58 ± 2.71
Female	18.38 ± 3.74	41.50 ± 4.73	10.97 ± 2.67
*p*-value	**< 0.001**	**< 0.001**	**< 0.001**
**District**			
Beirut	17.91 ± 3.73	41.21 ± 5.04	11.20 ± 2.67
Mount Lebanon	18.29 ± 3.91	41.59 ± 4.65	10.79 ± 2.72
North	18.02 ± 3.70	41.25 ± 4.63	10.59 ± 2.70
South	18.32 ± 4.08	40.96 ± 5.31	10.65 ± 2.62
Bekaa	17.83 ± 3.53	40.07 ± 4.84	10.23 ± 2.79
*p*-value	0.184	0.054	**< 0.001**
**Education level**			
Illiteracy	14.90 ± 5.30	38.24 ± 4.60	8.54 ± 3.62
Primary	16.19 ± 4.02	39.91 ± 5.79	10.07 ± 3.39
Complementary	17.07 ± 3.92	40.42 ± 4.70	10.43 ± 2.69
Secondary	17.85 ± 3.64	41.22 ± 5.09	10.79 ± 2.72
University	18.88 ± 3.59	41.74 ± 4.63	11.13 ± 2.47
*p*-value	**< 0.001**	**< 0.001**	**< 0.001**
**Marital status**			
Married	18.10 ± 3.81	41.27 ± 4.89	10.85 ± 2.68
Single (divorced/widowed)	17.93 ± 4.11	39.67 ± 4.98	10.07 ± 3.00
*p*-value	0.654	**0.001**	**0.004**
**Monthly salary**			
< 1000 USD	17.93 ± 3.87	40.85 ± 4.82	10.54 ± 2.89
1000–2000 USD	18.05 ± 3.80	41.38 ± 4.96	10.93 ± 2.58
> 2000 USD	18.82 ± 3.54	41.04 ± 5.12	11.04 ± 2.53
*p*-value	**< 0.001**	0.052	**0.001**
**Type of insurance**			
None	17.64 ± 3.71	40.62 ± 4.83	10.28 ± 2.80
National Social Security Fund (NSSF)	18.35 ± 3.80	41.48 ± 4.85	11.12 ± 2.67
Army	18.25 ± 3.70	41.76 ± 4.49	10.70 ± 2.66
Private	18.15 ± 3.85	41.01 ± 5.32	11.02 ± 2.42
Cooperative	19.37 ± 3.74	41.88 ± 4.96	10.92 ± 2.61
*p*-value	**< 0.001**	**< 0.001**	**< 0.001**

Numbers in bold indicate significant p-values

Higher knowledge was significantly but weakly associated with better attitude and practice, whereas a better attitude was significantly associated with better practice. Furthermore, a better PPC was significantly associated with better knowledge, attitude and practice respectively. Finally, a higher number of children and a higher household crowding index were significantly associated with lower knowledge, attitude and practice respectively (Table 3).

**Table 3:** Bivariate analysis of the continuous variables associated with the knowledge, attitude and practice scores

Variable	Knowledge	Attitude	Practice
Knowledge	1		
Attitude	0.171 ^c^	1	
Practice	0.152 ^c^	0.269 ^c^	1
Patient-physician communication	0.196 ^c^	0.284 ^c^	0.3^c^
Age	−0.02	−0.043 ^a^	0.028
Number of children	−0.071^c^	− 0.107 ^c^	− 0.047^a^
House crowding index	− 0.117 ^c^	− 0.03	− 0.054 ^b^

Numbers in this table correspond to the correlation coefficients of the Pearson correlation test; ^a^ *p* < 0.05; ^b^*p* < 0.01; ^c^*p* < 0.001; numbers without an asterisk correspond to non-significant associations

### Multivariable analysis

Multivariable analysis was performed in order to assess which variable would be more associated with the knowledge, attitude and practice. The practical application of multivariable statistics to a particular problem may involve several types of univariate and multivariable analyses in order to understand the relationships between variables and their relevance to the problem being studied.

A first linear regression, taking the knowledge score as the dependent variable, showed that female gender, having a higher education level vs illiteracy, a cooperative type of insurance vs no insurance and a better PPC were significantly associated with higher knowledge, whereas having an intermediate vs low monthly income was significantly associated with lower knowledge (Table 4, Model 1).

**Table 4:** Multivariable analysis

	Standardized Beta	Confidence interval	
		Lower Bound	Upper Bound
**Model 1: Linear regression taking the knowledge score as the dependent variable.**			
Gender (females vs males^a^)	0.041	0.010	0.321
Primary level of education vs illiteracy^a^	0.09	0.114	2.677
Complementary level of education vs illiteracy^a^	0.269	1.596	4.023
Secondary level of education vs illiteracy^a^	0.377	2.150	4.539
University level of education vs illiteracy^a^	0.557	3.026	5.376
Intermediate monthly income vs low income^a^	−0.049	− 0.673	− 0.077
Cooperative type of insurance compared to no insurance^a^	0.053	0.241	1.594
Patient-physician communication score	0.157	0.049	0.082
**Model 2: Linear regression taking the attitude score as the dependent variable.**			
Secondary level of education vs illiteracy^a^	0.077	0.266	1.432
University level of education vs illiteracy^a^	0.132	0.705	1.783
High monthly income vs low income^a^	−0.06	−1.229	−0.195
Cooperative type of insurance compared to no insurance^a^	0.046	0.074	1.956
Patient-physician communication score	0.281	0.124	0.170
Knowledge score	0.082	0.048	0.161
**Model 3: Linear regression taking the practice score as the dependent variable.**			
Primary level of education vs illiteracy^a^	0.124	0.422	2.208
Complementary level of education vs illiteracy^a^	0.199	0.641	2.341
Secondary level of education vs illiteracy^a^	0.245	0.713	2.390
University level of education vs illiteracy^a^	0.306	0.815	2.479
National social security funds compared to no insurance^a^	0.087	0.241	0.705
Private insurance compared to no insurance^a^	0.046	0.038	0.706
Patient-physician communication score	0.194	0.046	0.070
Knowledge score	0.07	0.022	0.079
Mount Lebanon compared to Beirut^a^	−0.051	−0.620	−0.048
North Lebanon compared to Beirut^a^	−0.06	−0.639	− 0.098
Single status (divorced/widowed) compared to married	−0.09	−1.125	− 0.020
Attitude score	0.206	0.091	0.136

^a^Reference group

A second linear regression, taking the attitude score as the dependent variable, showed that having a higher education level vs illiteracy, a cooperative type of insurance vs no insurance, a better PPC and better knowledge were significantly associated with better attitude, whereas having a high vs low monthly income was significantly associated with a worse attitude (Table 4, Model 2).

A third linear regression, taking the practice score as the dependent variable, showed that having a higher education level vs illiteracy, having insurance vs not, a better PPC, better knowledge and better attitude were significantly associated with better practice, whereas being single compared to married were significantly associated with worse practice (Table 4, Model 3).

## Discussion

Overall, this is the first national Lebanese study aiming at assessing parents’ KAP towards children’s vaccination. The major findings of our study are that having a higher level of education, a better PPC, and having insurance were associated with better KAP. Moreover, having a higher monthly income was associated with less knowledge and a negative attitude towards vaccination.

Our results showed that 757 (27.2%) had good knowledge towards children’s vaccination. A study done in Sri Lanka showed that 44.0% of the participants had above average, 9.2% had average and 46.8% had below average knowledge [34]. The majority of the participants (90.1%) thought that vaccinating their children is very important, did not delay or plan to delay a vaccine and 3.5% of the participants stated that they were not in favor of vaccination despite bringing their children to get vaccinated [34]. In a study done in Quebec, the majority of mothers had a low level of knowledge on vaccination, even those who intended to have their infant vaccinated as recommended [33]. In addition, 740 (26.8%) parents had good attitude towards vaccination. Fifteen percent of mothers had a score indicating a high level of vaccine hesitancy and one third had an intermediate level of vaccine hesitancy [33]. Finally, 910 (32.8%) parents had good practice towards vaccination in children. Findings in Quebec showed that 22.5% of mothers were not certain about their intention to vaccinate their child [33].

### Patient-physician communication

Our results showed that having a good communication with the physician led to better vaccination KAP. In previous research, a good communication between parents and pediatricians or physicians had proved to provide parents with better knowledge regarding vaccination [37]. In fact, having a poor communication with the health care provider will push parents to look into other sources that can be inaccurate [22]. In addition, a better PPC was linked to positive attitudes, in line with previous findings [19] that shed the light on the role of the health care provider in encouraging the parents in the direction of vaccination. Physicians play an important role in shaping parents’ beliefs by informing them about the benefits and safety of vaccines. Adding to that, studies have shown that having a good relationship with the physician built on trust will increase vaccination rates [38].

### Knowledge

Our study has shown that female gender having a primary, complementary, secondary and university levels of education in comparison with illiteracy, were significantly associated with better knowledge. On another note, having an intermediate monthly income compared to a low income is associated with reduced knowledge. Our results support the findings of previous research [15, 39] that stated mothers were more aware of the immunization schedule of their children compared to fathers. Also, previous research has talked about the association of a higher level of education with more knowledge about vaccination [40]. This might be due to the fact that being more educated allows a better communication with health care providers and less chances of acquiring wrong beliefs regarding vaccines [41]. Our results showed that having an intermediate monthly income was accompanied with less knowledge when compared to low income. This contradicts other studies [31, 42] that concluded that having a higher income provides more access to health care providers and physicians and more information about vaccines. This might be because families with higher income have access to good health care and feel secure and assured by offered vaccination programs without further investigation about the topic.

### Attitude

Additionally, having a secondary and university level of education and better knowledge are significantly associated with a better attitude. In contrast, having a high monthly income is associated with a worse attitude. This goes in agreement with previous results [39, 43] that demonstrated that parents with higher education showed positive attitudes towards vaccination mostly because these parents tended to understand more its importance. Educated parents seem to understand more the risks of infectious diseases and the benefits of vaccination in their prevention. Moreover, we found that parents with a high income showed more negative attitudes than those with a low income. Reaffirming previous results, having a high income is associated with less vaccination rates [14]. This might be because parents with a high income have the means to treat their children and live near health facilities. Some may also think that they can protect their children through less exposure and healthier lifestyles. But some studies also noticed that having a low income can be associated with a negative attitude since some parents with low income choose to spend money on other necessities [44].

### Practices

Finally, educated parents compared to illiterate parents, having an insurance through a national security fund or a private insurance and having better knowledge and attitude were significantly associated with better immunization practices. Previous studies done on the association between the level of education and the practices are contradictory. While some studies go in the same direction with our results to prove that having a high level of education was linked with better immunization coverage [15, 37, 38], others clearly state that having a higher level of education was associated with negative vaccination practices [14]. A study that was done in Lebanon in 2018 to evaluate vaccine coverage in children showed that children whose mothers were illiterate had lower vaccination rates than those with educated mothers [9]. Having a higher education is generally accompanied with more knowledge about the safety of vaccines and the severity of VPD. Additionally, having an insurance could be associated with higher vaccination rates because one of the reasons for vaccine hesitancy is the cost of vaccines [45], specially that in Lebanon we don’t not have universal health coverage. This was also shown in a previous Lebanese study where having a private insurance resulted in higher rates of vaccination [9]. At last, our results go with the literature to prove that having more knowledge about the efficacy of vaccines, and having a positive attitude towards vaccination and its benefits, all will increase vaccination rates [15, 34].

### Clinical implications

The decrease in vaccination rates is a problem that parents as well as physicians should be well aware of. The results of our study proved the importance of health care providers in shaping the opinion of parents and their vaccination practices. When dealing with parents, the pediatrician or other physician should be able to properly communicate the importance of vaccines, their safety, and the consequences of noncompliance with the schedules.

### Limitations

Being a cross-sectional study, our research faces some limitations. Causal relationships could not be observed with this type of study. Also, our study type is subject to some biases. A non-differential bias could have occurred since parents may under or overestimate a question. Another limitation is the possibility of occurrence of a recall bias, especially in parents who did not have vaccination cards. A selection bias might be possible because of the refusal rate and since no comparison could be done between parents who refused and those who accepted to enroll in this study. Furthermore, although our analysis took into account several confounding factors, other confounding variables might not have taken into consideration predisposing us to a residual confounding bias. A social desirability bias is also possible since survey respondents tend to answer questions in a manner that will be favorably viewed by others. Finally, the scales used in this study have not been validated prior to data collection.

## Conclusion

Vaccination is one part of public health that saves millions of lives every year. Nowadays, multiple outbreaks are happening worldwide. Lebanon is no exception, with the reappearance of measles and mumps since the arrival of refugees. The methods used to collect data and the weighting done in the statistical analysis allow generalization of these results to the whole population. Our study sheds light on the importance of the physician in improving the KAP of parents towards their children’s immunization. More emphasis should be put on the importance of this relationship and the need to consult a physician. It also calls attention to the need to guide parents, especially those with lower levels of education, on the importance of vaccination. It is important to identify new strategies to highlight the need for immunization and educate parents about the importance of vaccines on an individual and public health level. Future studies are needed to ensure that vaccination coverage is increasing in Lebanon with time and that appropriate actions are being taken to a better parental guidance.

## Data Availability

There is no public access to all data generated or analyzed during this study to preserve the privacy of the identities of the individuals. The dataset that supports the conclusions is available to the corresponding author upon request.

## Abbreviations

KAP: Knowledge, attitude and Practice
PPC: Patient-physician communication
VPD: Vaccine preventable diseases
MCV: Measles containing vaccine
SD: Standard deviations
